# Hydration of Hybrid Cements at Low Temperatures: A Study on Portland Cement-Blast Furnace Slag—Na_2_SO_4_

**DOI:** 10.3390/ma15051914

**Published:** 2022-03-04

**Authors:** Shiju Joseph, Özlem Cizer

**Affiliations:** Materials and Construction, Department Civil Engineering, KU Leuven, 3001 Brussels, Belgium; ozlem.cizer@kuleuven.be

**Keywords:** hydration, microstructure, activation, sulfate, phase assemblage

## Abstract

Replacement of Portland cement with high volumes of blast furnace slag is known to negatively affect the early-age properties of concrete, particularly at low temperatures. In this study, the effectiveness of Na_2_SO_4_ on the mechanical properties, hydration kinetics and microstructure development of a commercial CEM III/B (~69% slag) is investigated at 10 and 20 °C. Na_2_SO_4_ enhances compressive strength at both 10 and 20 °C, and at both early (1 and 7 days) and later ages (28 and 90 days). QXRD shows an increase in the degree of alite hydration at 1 day with Na_2_SO_4_ addition, while the degree of clinker and slag hydration is similar for all the systems from 7 to 90 days. An increase in ettringite content is observed at all ages in the systems with Na_2_SO_4_. Microstructure and pore structure shows densification of hydrates and reduction in porosity on addition of Na_2_SO_4_.

## 1. Introduction

Ground granulated blast furnace slag or commonly denoted as blast furnace slag or just slag, is a by-product from the iron industry, that is used to replace Portland cement (PC) to enhance the performance of concrete and gain important economic and environmental benefits [1,2,3,4,5,6,7]. Due to its highly amorphous Ca-Si-Al-Mg glassy phase [1,2], slag is one of the most reactive supplementary cementitious materials (SCM) used in blended cements, and commonly presents *latent hydraulic* behavior. Owing to its high reactivity (compared to other SCMs), high calcium content and comparable specific surface area (after grinding) in comparison to PC, standards permit very high replacement levels for slag in cement. While up to 35% replacement of PC by slag is permitted in CEM II, the permissible replacement levels may reach 95% in CEM III according to EN 197-1, provided it meets the required quality criteria. It is estimated that around 300–360 million tonnes of slag are being produced each year globally, and this is almost entirely used for the production of blast furnace slag cements [8]. Although this is less than 1/10th of the ~4 billion tonnes of total cement produced worldwide [9], this quantity is still large enough to be worth the investment of resources to further improve its performance in concrete.

The worst issue associated with the high replacement levels of PC with slag, or any SCM in general is poor mechanical performance at early ages, and at low curing temperatures. Berodier and Scrivener [10] reported that during the initial day of hydration, blast furnace slag does not react chemically and only provides additional surface area to enhance PC hydration by filler effect [11,12,13]. This translates to slower strength development at early ages with increasing slag replacement levels, even though this is largely compensated at later ages [14,15,16].

Curing temperature has a significant effect on the kinetics of slag reaction and strength development in slag blended cements. Although low curing temperature significantly reduces the reactivity and strength development of plain PC systems, this is even more drastic in blended cements with increasing replacement levels of slag [17,18,19,20]. Although this is generally the case for all SCMs upon replacement with PC, only slag is replaced at such high ratios (60–90%). The reason for the higher impact on curing temperature for SCMs can be attributed to the higher apparent activation energies of SCMs compared to PC [21,22,23,24]. Based on the Arrhenius equation, the increase in reaction rate is higher when the activation energy is on the higher side.

As the alkalinity of the pore solution significantly affects the reactivity of SCMs, a possible route is to enhance the hydration of SCM using alkali activation. Although strong activators, such as NaOH, can significantly boost early-age hydration, the later-age mechanical properties are largely compromised, primarily due to their negative effects on PC hydration [25,26,27,28]. The high pH of such activators also makes it less feasible for general purposes due to the associated health risks. These issues make Na_2_SO_4_ attractive as it has a high solubility (~0.98 M), near neutral pH, and low enthalpy of solution [29]. Other additions (along with Na_2_SO_4_), such as sodium gluconate [30], calcium aluminate cements and potassium aluminate sulphates [31] are found to significantly boost the compressive strength at later ages.

Compared to fly ash blended cements, the published literature on the Na_2_SO_4_ activation of blast furnace slag blended cements are relatively limited. Several researchers found an increase in the early-age strength with the addition of Na_2_SO_4_ in slag blended cements, while the later-age strength did not significantly change, or was only marginally reduced [27,31,32,33,34]. On the other hand, some studies suggest an increase in both early- and later-age strength with the addition of Na_2_SO_4_ [35,36]. Combination of Na_2_SO_4_ with other additives such as alum [31], calcined gypsum [32] and sodium gluconate [30] was also found to further improve the mechanical properties. Previous studies have investigated the use of Na_2_SO_4_ to activate the slag in an alkali activated system without PC addition, and this was found to have very low performance at ambient temperatures, particularly at early ages [37,38].

Nevertheless, in hybrid cements with PC, slag and Na_2_SO_4_, previous studies were carried out only at ambient temperatures, and the effect at low curing temperature is not known yet. In this study the effect of Na_2_SO_4_ activation on hydration of commercial CEM III/B 42.5 N is investigated at 10 and 20 °C. The objective of this study is to investigate whether we can compensate the slow reaction rate of blended cements at lower temperature with Na_2_SO_4_. The compressive strength development of mortar, kinetics of clinker and slag hydration, hydrate phase assemblage and microstructural development are investigated to unveil the effect of Na_2_SO_4_ activation on low temperature hydration reactions.

## 2. Materials and Methods

### 2.1. Materials

A commercial blast furnace slag cement, CEM III/B 42.5 N, and a technical grade Na_2_SO_4_ were used in this study. The particle size distribution of the cement was characterized using laser diffraction after dispersion in isopropanol prior to measurement. Particle fineness was also characterized using the standard Blaine technique. The oxide composition of the cement was determined using a wavelength-dispersive X-ray fluorescence (XRF) spectrometer. The mineralogical composition was determined using quantitative X-ray diffraction. The amount of slag present in cement was further verified using the selective dissolution technique.

### 2.2. Sample Preparation

The experimental study was performed on paste and mortar specimens. The hydration, phase assemblage and microstructure were characterized at paste scale and the strength development was monitored on mortar scale (Table 1). All samples were prepared in a temperature-controlled room at 20 °C. Na_2_SO_4_ solution of strength 0.75 M was used based on a previous study on fly ash [39]. The water to cement ratio (*w*/*c*) was kept constant at 0.5, which resulted in a higher solution to cement ratio (S/c) of ~0.55 for mixes with Na_2_SO_4_ solution. In this definition, cement includes all the binder mass including the Portland cement and blast furnace slag, and Na_2_SO_4_ is considered to be the part of the solution.

Pastes were prepared by means of a vertical high shear mixer at 1000 rpm for 2 min. They were then sealed in plastic vials and cured at 10 °C and 20 °C up to 90 days. Mortar samples were prepared following EN 196-1 using the norm sand in a Hobart mixer, and then cast into 40 × 40 × 160 cm moulds. The mortar prisms were sealed using plastic film to prevent evaporation and cured at 10 °C and 20 °C up to 90 days. The compressive strength development was monitored by testing the mortar bars at 1, 7, 28 and 90 days in accordance with EN 196-1 at a loading rate of 2 mm/min.

### 2.3. Isothermal Calorimetry

Heat release from 45 min after mixing (which also takes into account the time required for stabilization of signal in calorimeter after placing) until 7 days was measured using a TAMair 8 channel isothermal calorimeter at 10 and 20 °C. During measurements at 10 °C, the calorimeter was kept in a climate-controlled room at 10 °C and <60% RH to avoid condensation. Around 7–8 g paste was transferred into an ampoule before placing in the calorimeter. The reference cell was filled with an ampoule filled with water having approximately equal thermal mass of samples.

### 2.4. Hydration Stoppage

Hydration of the pastes was arrested after 1, 7, 28 and 90 days of sealed curing using a freeze drier for 2 h at 0.025 mbar pressure after crushing them with a mortar and pestle. For MIP and electron microscopy, hydration was stopped using solvent exchange method. Samples were cut into a disc of ~3–5 mm and immersed in isopropanol for 7 days. The solution was replaced with new isopropanol after 1 and 3 days. Afterwards, the samples were dried in a vacuum desiccator.

### 2.5. TGA

Thermogravimetric analysis (TGA) was used to quantify the total bound water content by measuring the mass loss from room temperature up to 600 °C. TGA was carried out on NETZSCH STA 409 PC, at 10 °C/min heating rate under nitrogen flow. Typically, around 20–40 mg of the sample was used for each measurement. The portlandite content was quantified using the tangent method [40] and is reported as g/100 g anhydrous cement [41].

### 2.6. XRD Measurement

X-ray diffraction (XRD) was carried out on hydration-arrested pastes ground to powder using a Bruker D2 phaser diffractometer with Cu Kα radiation (λ = 1.54 Å). The samples were scanned over the range of 5–55° 2θ. Anhydrous cement was characterized using the internal standard method, mixing 20% of zincite. Additional measurements without internal standard were also conducted to aid quantification of the degree of hydration. For the quantitative analysis of the pastes, corundum was used as an external standard. Step size of 0.02° 2θ and 0.3 s/step was employed. After 90 days of hydration, additional XRD measurement was performed on a freshly cut disc to further compare the effect of hydration stoppage on phase assemblage. The fresh disc of 3–5 mm thickness was cut from a cylinder and was gently polished with a sandpaper to flatten the surface. A slower XRD scan of 0.6 s/step was conducted to gain higher resolution of the signal.

### 2.7. Rietveld-PONKCS Analysis

The quantification of the phase composition was determined using the Rietveld method in Topas (Academic) software. Chebyshev polynomial with three background (bkg) parameters along with an 1/X term was used as the background function. The number of bkg parameters were limited to ensure that the amorphous humps from slag and C-S-H were not overfitted. Further, a fundamental parameter approach [42] was used and corrections for specimen displacement were conducted, while 5–8° 2θ scan was omitted. For the known crystal structures, lattice parameters and crystallite size were refined while atomic parameters were not refined, as per the recommendation of [43]. Lattice parameters were not allowed to deviate more than 1% from the literature values. Corrections for preferred orientation were conducted for alite M3, gypsum, anhydrite and portlandite.

To quantify the degree of hydration of the amorphous slag in cement paste, the “partial or not known crystal structure” method, PONKCS, was used [44]. C-S-H structure developed by Snellings [45] for hydrated white cement was used without any further refinement. For modeling the diffraction profile of an *amorphous hump* of slag, a set of pseudo Voigt peaks was used and was fitted on the anhydrous slag-cement. This was based on the assumption that all the contributions of signals from non-crystalline phases were from the slag. For capturing the profile of slag, two broad peaks at d value of 2.893 and 1.926 were used. The quantification of all phases were carried out using the external standard method [46]. The scale factor of the slag phase was compared with the scale factor of the anhydrous cement, considering the mass attenuation coefficients (MAC) and the G-factor determined from the external standard [46]. The MAC of the samples was calculated based on the oxide composition of cement determined from X-ray florescence (XRF), and bound water content determined from TGA. The expected error for the crystalline phases is 0.5 wt% and for the degree of hydration of amorphous slag is around 7%.

### 2.8. Electron Microscopy

Samples after hydration stoppage were embedded in resin under vacuum for 24 h. These were progressively polished with P1200 through to P4000 SiC papers with the aid of ethanol and using 3 µm and 1 µm diamond spray (oil-based). Electron microscopy study was carried out on FEI XL 30 FEG with EDAX energy dispersive spectrometry (EDX) detector. The accelerating voltage was 15 kV and working distance was around 10 mm with field emission gun (FEG) as electron source. Images were captured on backscattered electron (BSE) mode and EDX point analysis (~50 measurements per sample) was done on both anhydrous grains and hydration products.

### 2.9. MIP

Mercury intrusion porosimetry (MIP) was carried out on paste samples after solvent exchange using Micromeritics AutoPORE IV 9500. The pressure was progressively increased to 30,000 psia (~207 MPa). The contact angle of mercury was assumed to be 130° and the surface tension to be 0.485 N/m.

### 2.10. Selective Dissolution

Selective dissolution was carried out to evaluate the slag content following the recommendation of RILEM TC 238-SCM [47], using triethanolamine (TEA), EDTA and diethylamine (DEA). A mixture of 250 mL TEA, 500 mL distilled water, 93 g EDTA and 173 mL DEA was prepared and brought to 1000 mL by adding distilled water. Then 50 mL of this solution was diluted to 800 mL with distilled water. Then 0.5 g of cement was added and stirred for 2 h. Then it was filtered, and the residue was washed 5 times with 10 mL of distilled water. The residue was weighed after drying at 105 °C for 1 h. The results from selective dissolution of hydrated cements were discarded as XRD measurement on the selective dissolution residue showed signals of C-(A)-S-H.

## 3. Results

### 3.1. Characterisation of Cement

**Figure 1:**
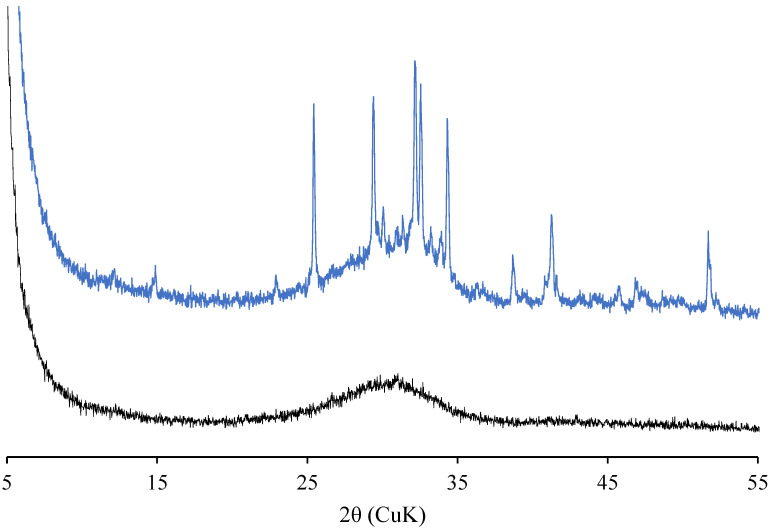
XRD diffractograms of CEM III before (blue) and after (black) selective dissolution. The sharp peaks correspond to the anhydrous clinker phases in the cement and the broad peak correspond to the amorphous glassy phase of slag.

Chemical composition, mineral phases and physical properties of CEM III/B 42.5 N are presented in Table 2. Alite is the major phase in clinker, with 18% out of 24.3% crystalline clinker phases. The amorphous content in the cement was quantified to be 69.1%, which can be assumed to have originated from the slag, as commercially produced unhydrated Portland clinker rarely contains amorphous fraction [48]. This content was also validated by the percentage of residue after selective dissolution, which was 69.4%. The XRD diffractograms before and after selective dissolution are shown in Figure 1. The residue after selective dissolution was devoid of any crystal peaks. A total percentage of 3.1% CaSO_4_ is present as bassanite and anhydrite, while peaks of gypsum are not observed.

Furthermore, the oxide composition and elemental ratios of the slag as determined by SEM-EDX analysis are shown in Table 3. These points were selected from hydrated pastes, and EDX point analysis was performed on the anhydrous grains. Those points were confirmed to be slag particles, based on the grey level intensity, the morphology and the presence of magnesium content. The measured composition seems to be very similar to typical slag compositions available in western Europe [1,2,49].

### 3.2. Compressive Strength

Mortar samples with CEM III/B only are denoted by III, and those with Na_2_SO_4_ added are denoted by NS. Additional denoting with 10 and 20 indicates the temperature of curing in °C. The flow table value increases by around 12% with the addition of Na_2_SO_4_ (Table 1). This is in agreement with the previous study [39]. The effect of Na_2_SO_4_ solution in increasing mortar flow implies that similar flow values can be achieved by reducing the *w*/*c* ratio.

**Figure 2:**
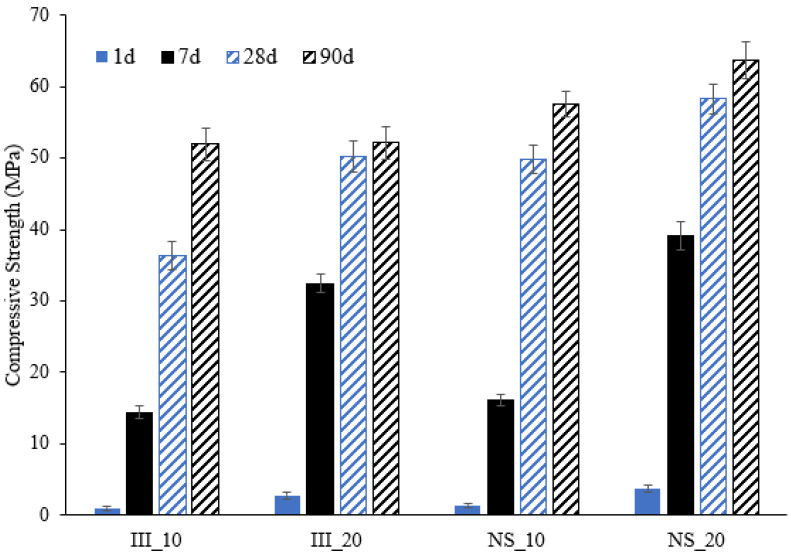
Compressive strength development of the mortars from 1 day until 90 days cured at 10 and 20 °C. Notation: III—mixes with water and NS—mixes with Na_2_SO_4_ solution. 10 & 20 represent the respective curing temperatures used.

Figure 2 shows that at both temperatures, all mortar mixes developed compressive strength up to 90 days. The addition of Na_2_SO_4_ in NS mortar mixes boosted the compressive strength at all ages and at both 10 and 20 °C when compared with III mixes. The NS mortars at 7 days yielded an increase of 10% and 20% at 10 °C and 20 °C respectively. Similarly, improvements of 37% and 10% strength were recorded at 28 and 90 days respectively for 10 °C, while at 20 °C, this increment was presented around 16% and 10% respectively.

### 3.3. Hydration Kinetics

**Figure 3:**
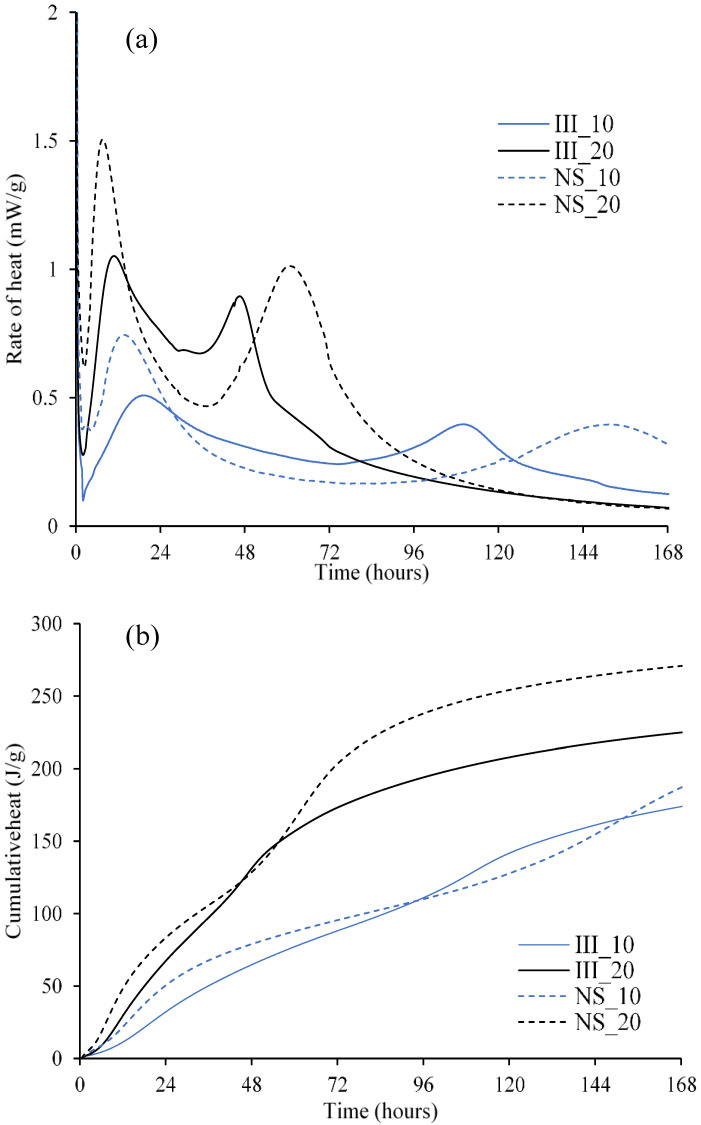
Rate of heat release (**a**) and cumulative heat (**b**) measured from isothermal calorimeter normalized to per gram of cement.

The rate and cumulative heat of hydration of the studied mixes are shown in Figure 3a,b respectively. Two distinct peaks are clearly captured in the hydration rate curves of all the mixes. The intensity of the first peak increases with both an increase in temperature and the addition of Na_2_SO_4_. This indicates a higher degree of reaction of alite at early ages. This is further confirmed with the quantitative XRD analysis (Table 4), as the degree of hydration of alite after 1 day of hydration was significantly enhanced both with the addition of Na_2_SO_4_ [50] and the increase in the curing temperature.

The second peak of hydration in the reference mix (without Na_2_SO_4_) appears much earlier and sharper at 20 °C (III_20) when compared to that at 10 °C (III_10). This peak overlaps with the deceleration period of alite hydration and a narrower peak is seen at 20 °C compared to 10 °C. On the other hand, when Na_2_SO_4_ is added, this second hydration peak is delayed at both temperatures. At 7 days, the second peak is still in progress in the case of the mix with Na_2_SO_4_ cured at 10 °C (NS_10), while all other mixes have already past the second peak. This can explain considerably higher improvement in strength for NS_10 after 7 days (around ~8–10 days) compared to others as the rate of hydration was higher during this period.

The strength trends at early ages (1 d and 7 d) resurface in the trends related to cumulative heat release (Figure 3b). Total heat release was most affected by the increase in temperature. Low heat release at 1 d was due to rather lower alite content (~18%) compared to Portland cement (50–70%) which resulted in a low compressive strength at 1 d. At 20 °C, NS_20 releases higher heat consistently except for a short duration around 2 days, which overlaps with the second hydration peak. Beyond that, it releases significantly higher heat. At 10 °C, NS_10 yields lower heat release between 4 and 6 days, again corresponding to the peaks of hydration. This would further translate into lower strength in this period.

**Figure 4:**
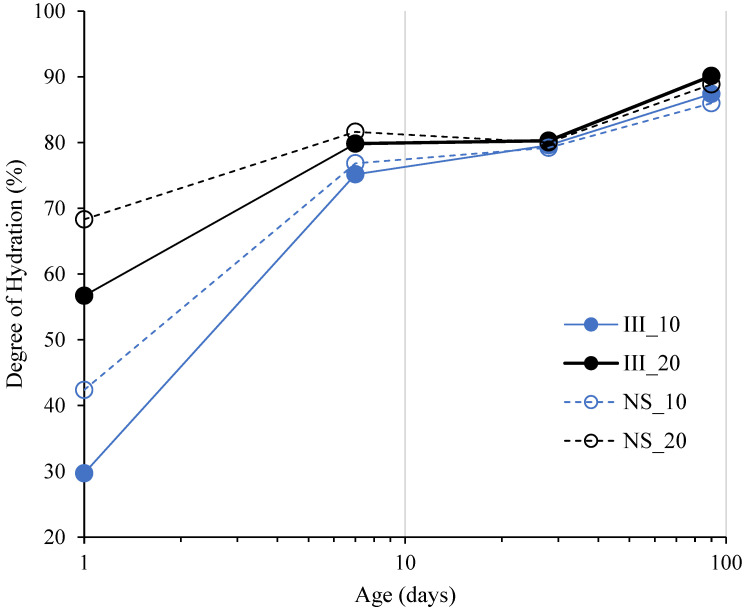
Degree of hydration of clinker in the studied mixes.

Figure 4 shows the degree of hydration of clinker and Table 4 reports the degree of hydration of the individual clinker phases along with the unhydrated mass per 100 g of anhydrous cement determined with XRD/Rietveld analysis. Negative values appear in the table due to a combined effect of the error expected from the XRD quantification, the low contents of the phases and low degree of hydration. The degree of hydration of clinker (DoH_clinker_) was calculated from the degree of hydration of the individual phases taking into account the initial mass percentage. When the measured degree of hydration of a particular phase was negative, this was assumed to be zero in the calculation of the degree of clinker hydration.

After 1 day of hydration, there were considerable variations in the DoH_clinker_ of the studied systems. The DoH_clinker_ was clearly enhanced both by temperature and Na_2_SO_4_ addition. At 7 days, a similar trend was visible, but the variations between the systems are much lower than that at 1 day. At 28 days, DoH_clinker_ was similar for all the systems, and at 90 days, the presence of Na_2_SO_4_ had a slightly negative effect. This trend was not similar to what was observed in the mortar strength at 28 and 90 days. This indicates that the contribution of clinker hydration to strength development was high at early ages, and at later ages, other factors are also at play.

The degree of clinker hydration was significantly controlled by the degree of alite (C_3_S) hydration of (Table 4). This was expected, due to the high quantities of alite in the clinker, which reacted almost completely. At early ages, the degree of alite hydration increased with the increase in temperature and addition of Na_2_SO_4_. However, not much increase as a response to these factors was recorded after 7 days. The temperature and Na_2_SO_4_ did not seem to influence the degree of alite hydration at 90 days.

Most of the C_3_A reaction happened from 1 d to 7 d. Slightly lower degrees of hydration for C_3_A and C_4_AF at 90 days were observed in NS systems. C_3_A is found not to be fully hydrated even after 90 days in NS_10. Some XRD signal reflections for C_3_A were also observed in NS_20. Even though the difference in the absolute values of the C_3_A was very small, this phenomenon correlates well with the literature in the presence of Na_2_SO_4_ [27,50,51].

The degree of C_2_S reaction seems to be negligible or within the error range of the experiment for all the studied samples until 28 days of hydration. However, there was a considerable increase in DoH of C_2_S by 90 days. The other slow reacting phase, C_4_AF, was found to be more reactive than C_2_S. For both C_4_AF and C_2_S, the differences between the degree of hydration were within the experimental error. Nevertheless, the increase in DoH_clinker_ after 28 days is attributed to the hydration of C_2_S and C_4_AF.

**Figure 5:**
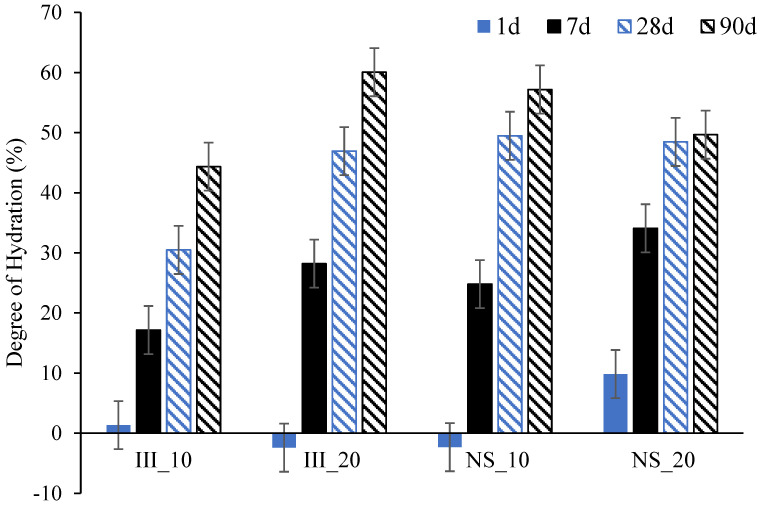
Degree of hydration of slag from XRD-PONCKS method.

Figure 5 shows the degree of hydration of slag (DoH_slag_) at 1, 7, 28 and 90 days obtained from XRD-PONCKS. DoH_slag_ was negligible at 1 d for all mixes except for NS_20. At 7 days, it increased both by temperature and Na_2_SO_4_ addition. At 28 days, DoH_slag_ was almost the same for all mixes, except III_10 which was slightly lower than that of others. A striking finding was that Na_2_SO_4_ addition enhanced DoH_slag_ at 10 °C up to 90 days, when the value was 13% higher than its reference counterpart (III_10). At 20 °C, its impact was mostly seen within the early ages up to 7 days.

### 3.4. Phase Assemblage

**Figure 6:**
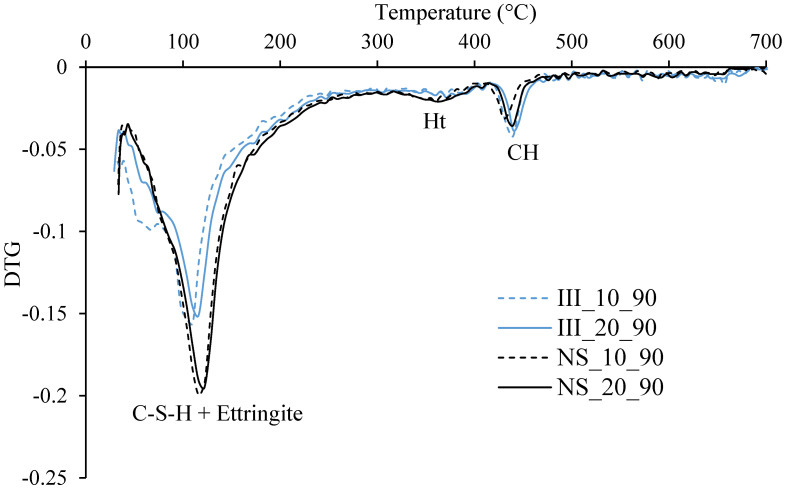
DTG of all mixes after 90 d hydration. Ht—hydrotalcite, CH—portlandite.

**Figure 7:**
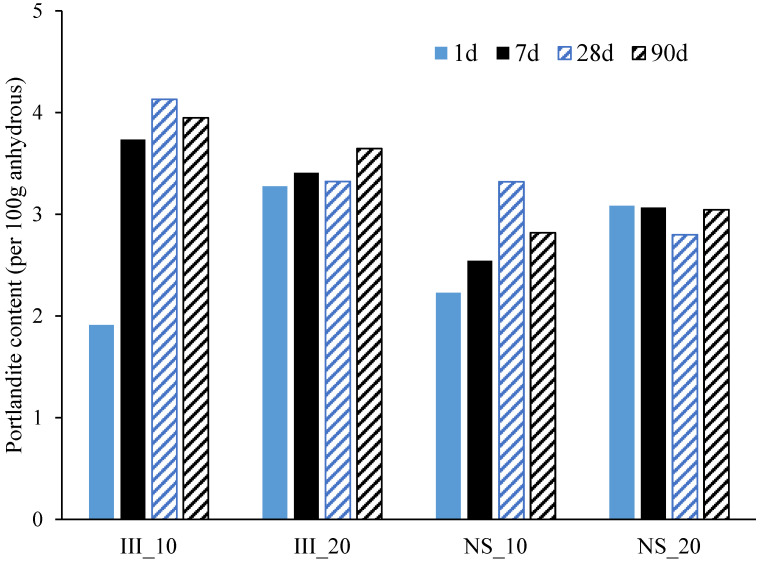
Portlandite content normalized to g/100 g anhydrous cement, determined from TGA-tangent method.

The plot of differential thermogravimetric analysis from TGA is shown in Figure 6 for samples after 90 days of hydration. The main mass loss was around 70–150 °C indicating the decomposition of C-(A)-S-H and ettringite. The mass loss during this peak was significantly higher for NS samples indicating higher ettringite and/or C-(A)-S-H content. A small mass loss at around ~350 °C indicated the decomposition of hydrotalcite (Mg_6_Al_2_O_9_CO_3_.12H_2_O) [52]. Mass loss associated with portlandite was also clearly visible at around 420–460 °C and was plotted in Figure 7. The amount of portlandite content was consistently low at around 2 wt%–4 wt%, and remained almost consistent after 1 day of hydration. The low portlandite content was expected due to low clinker content and high amounts of blast furnace slag. NS samples had lower portlandite contents, particularly at 10 °C and 90 days, when compared to samples without Na_2_SO_4_. This supports higher DoH_slag_ in NS_10 compared to III_10 and implies slag-portlandite reaction forming C-(A)-S-H. Further, the difference would be also partially associated with the reaction of Na_2_SO_4_ with portlandite to form gypsum and NaOH.

**Figure 8:**
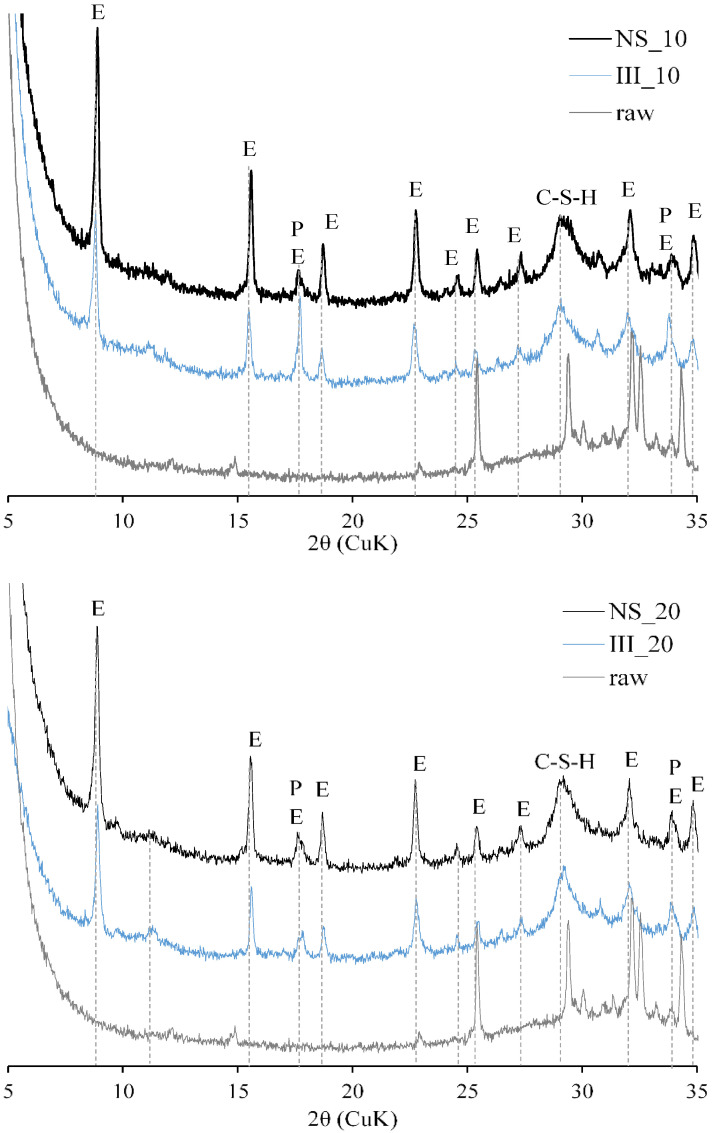
Qualitative XRD of 90 day old fresh samples without hydration stoppage. E—ettringite, P—portlandite, Ht—hydrotalcite.

Figure 8 qualitatively compares the XRD patterns of the samples at 90 days of hydration on freshly cut discs without hydration stoppage procedure. The clinker phases were almost fully reacted in all samples. The main reflections were associated to ettringite and C-(A)-S-H. Higher signals from ettringite reflections are clearly visible in the NS sample. A minor peak associated with hydrotalcite and portlandite peaks are also visible. No crystalline peaks associated with sodium substituted AFm phase (U-phase) [53,54] was found. Also, peaks corresponding to thenardite or mirabilite were not identified in any samples at 90 days, with or without hydration stoppage. Although reflections from thenardite were found from samples with Na_2_SO_4_ after 1 day of hydration, and after hydration stoppage. This is logical as Na_2_SO_4,_ which was completely dissolved in pore solution, will precipitate upon drying.

**Figure 9:**
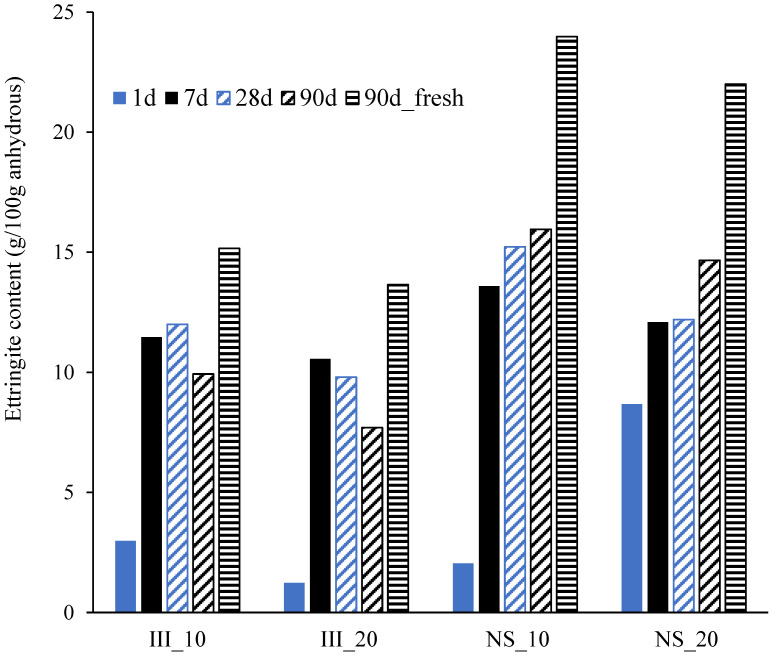
Ettringite content determined from QXRD (g per 100 g anhydrous cement). 90 d_fresh indicates the quantitative result on the sample without hydration stoppage.

Figure 9 plots the ettringite content quantified from XRD/Rietveld analysis at different ages. Only for the sample cured for 90 days, quantification was done on both freshly sawn disks and powdered samples after hydration stoppage for comparison purposes, while for other ages XRD measurements were only performed after hydration stoppage as a common protocol to suppress the progress of hydration. When the samples with and without hydration stoppage were compared, it was clearly seen that hydration stoppage underestimated the ettringite content quite significantly, which is in agreement with the previous studies [55,56,57]. For all samples, there was a significant increase in the ettringite content from 1 day to 7 days. While comparing samples without Na_2_SO_4_ (III_10 and III_20), the ettringite content seemed to increase for III_10 up to 28 days and decreases at 90 days, while it increases till 7 days and then kept on decreasing at 28 and 90 days for III_20. This indicates the formation of AFm phases after sulphates were fully consumed although this was not evident from XRD, possibly due to lack of long-range order [58]. On the other hand, in the NS samples, the ettringite content kept on increasing until 90 days.

**Figure 10:**
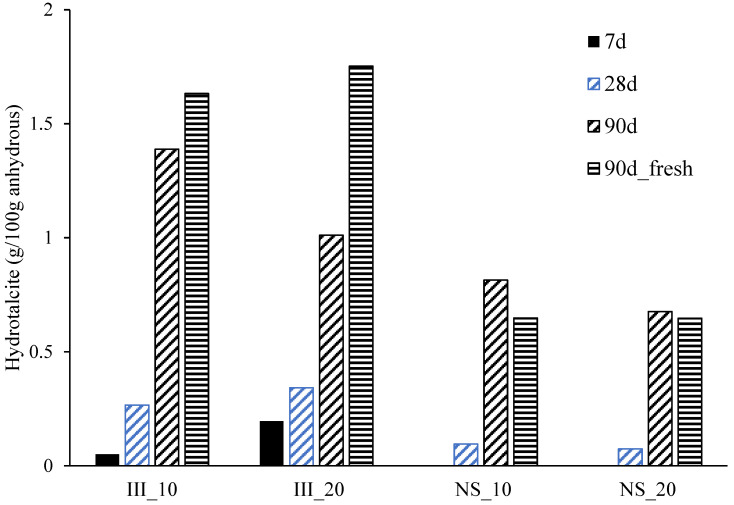
Hydrotalcite content determined from QXRD (g per 100 g anhydrous) from 7 to 90 days. At 1 day, none of the samples measured had traces of hydrotalcite. The denotation 90 d_fresh indicates the quantitative result on the sample without hydration stoppage.

Figure 10 reports the hydrotalcite content measured from XRD at different ages. After 1 day of hydration, none of the samples showed any signal reflection associated with hydrotalcite. At 7 days, samples without Na_2_SO_4_ had some detectable amounts of hydrotalcite, while NS samples did not have any. By 28 days, hydrotalcite was visible in all samples and became more available by 90 days. The amount of hydrotalcite was higher in the samples without Na_2_SO_4_ at all ages. Furthermore, no systematic differences were observed in the quantities measured in fresh samples and after hydration stoppage and the differences remained within the experimental error (0.5 wt%).

Table 5 reports the elemental ratios of the hydrate phases from SEM-EDX point analysis after 28 days of hydration. The main hydrates, C-(A)-S-H and ettringite were considerably intermixed: hence, the ratios corresponded to the overall hydration products. The Si/Ca ratio was higher in NS samples. This corresponded to the Ca/Si ratio of 1.85 in the samples without Na_2_SO_4_, and 1.69 in the samples with Na_2_SO_4_. This implies a higher degree of slag reaction and substitution of Ca with Na. Na/Ca ratios were also higher in NS samples as expected. Similarly, S/Ca ratio increases with Na_2_SO_4_ addition due to higher sulphates provided. Mg/Si ratios are also higher in NS, indicating a higher degree of slag reaction. Note that hydrotalcite was lower in NS mixes, which could mean that there is a higher incorporation of Mg in C-(A)-S-H. There were no significant differences in Al/Ca or Al/Si ratios between the samples.

### 3.5. Microstructure

**Figure 11:**
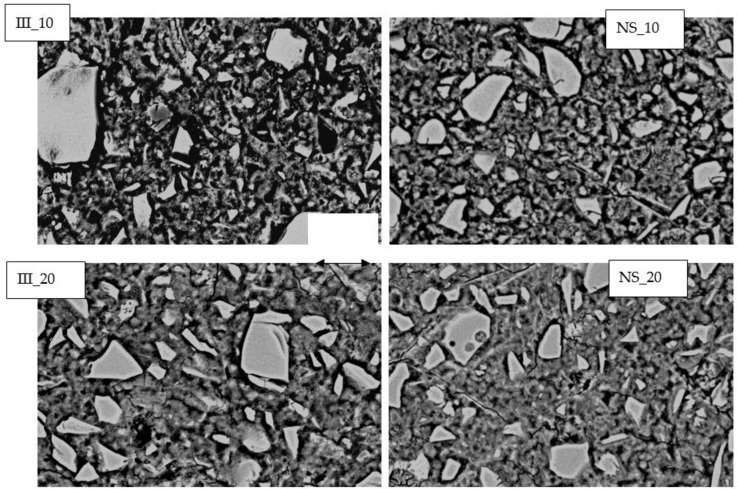
BSE Image of the mixes studied after 28 days of curing.

Figure 11 shows the microstructural slices for all the samples after 28 days of hydration captured on polished samples using SEM-BSE. Most of the unhydrated grains were found to be unreacted slag particles. A small gap between the hydrates and unhydrated slag grains, termed “Hadley grains” [59,60] were visible in all the samples. Both increase in temperature and addition of Na_2_SO_4_ contributed to densification of the microstructure. The addition of Na_2_SO_4_ is known to reduce the bulk density of C-S-H by transforming them into divergent bundles of fibrils [27,51].

**Figure 12:**
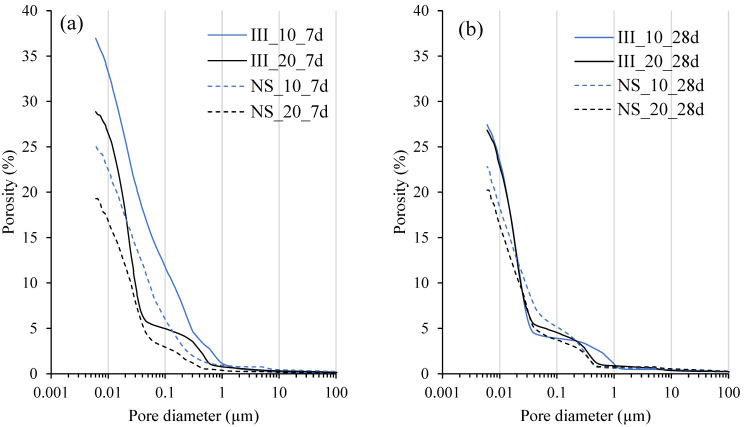
Pore size distribution of the samples determined using MIP after: (**a**) 7 days and (**b**) 28 days; of hydration.

Figure 12 shows the pore structure of the pastes measured with MIP after 7 and 28 days of hydration. At 7 days, curing temperature and the addition of Na_2_SO_4_ (NS_20) were favorable to reduce the overall measurable porosity. A considerable pore refinement is also evident from the results. By 28 days of hydration, samples cured at 10 and 20 °C without Na_2_SO_4_ had very similar microstructure, while a slight reduction in the porosity was seen in NS_20 compared to NS_10. Nevertheless, at the same curing temperature, NS samples had a lower porosity than their reference counterparts.

## 4. Discussion

### 4.1. Second Peak in Isothermal Calorimetry

**Figure A1:**
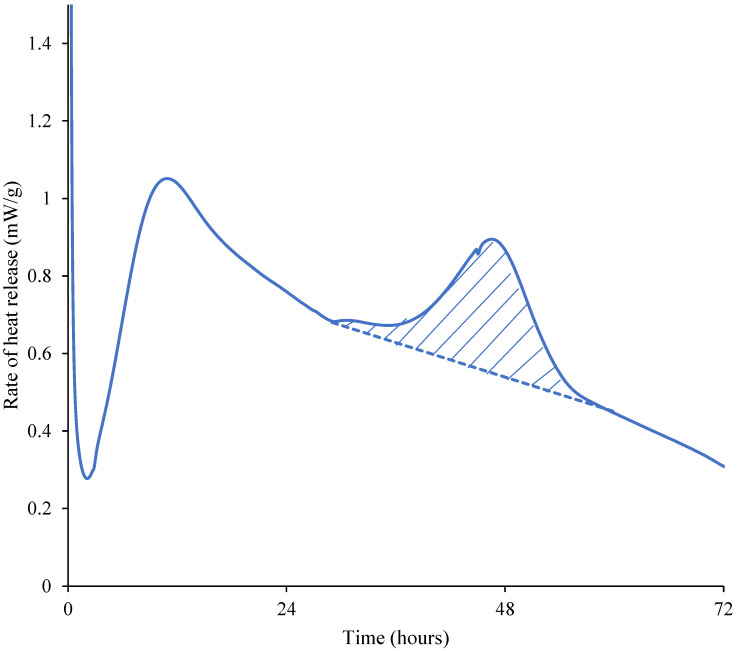
Method adopted to quantify the second hydration peak. The cumulative heat released from the second peak is calculated from the area of the shaded portion.

Richardson et al. [61] showed that by varying the gypsum content in PC-slag blended systems, the second peak from isothermal calorimetry (third peak if the initial heat release due to the fast dissolution is also to be considered) is associated with the reaction of aluminate. The addition of an alkaline activator such as Na_2_SO_4_ boosts the slag hydration, and it is common for alkali activated slag systems to have a hydration peak around 3–7 days. The area under the second peak (Figure 3a) was calculated using a similar approach that is used to measure the portlandite content from TGA results using tangent analysis (an example is shown in Appendix A, Figure A1). This method provides a rough estimation of the area under the second peak by subtracting the tail of the first peak of hydration. In the NS samples without Na_2_SO_4_ activator, the calculated heat release from the second peak was 13.5 J/g-_cement_ for III_20 and 16.4 J/g-_cement_ for III_10. The area under both peaks, considering the large error associated with the crude approach, appears to be similar. These peaks should be attributed to the main peak of C_3_A hydration [59,62,63].

In the NS samples activated with Na_2_SO_4_, the second peak appears to be delayed with respect to those ones without Na_2_SO_4_. On the other hand, the area under the peak is significantly higher: 58.9 and 45.0 J/g-_cement_ for NS_20 and NS_10 respectively. It should be noted that these values are only till 168 h (7 days) and the second peak of NS_10 was not completed by 168 h. These higher values indicate that this peak cannot be purely associated with C_3_A hydration as the mass percent of C_3_A reacted between 1 day and 7 days with Na_2_SO_4_ was even lower than those without. The theoretical maximum heat release, assuming all the C_3_A reacted during this period was around ~10–22 J/g-_cement_ even after considering the quantification error from Rietveld analysis. Thus, in NS samples, the second peak should be attributed to (at least partially) enhanced slag hydration. Figure 5 further shows an increase in the degree of hydration of slag by 7 days in NS samples. It is not clear from the current results if the contribution from the slag was mainly from Al_2_O_3_ phase in the slag.

### 4.2. Enthalpy of Slag Hydration

The degree of hydration of slag can be estimated from isothermal calorimetry, provided that the degree of hydration of clinker phases and the enthalpy of slag hydration are known. Based on the XRD results (Table 4), the heat released from the individual clinker phases was calculated based on the enthalpies of reactions reported in literature: 517 J/g (C_3_S), 262 J/g (C_2_S), 1674 J/g (C_3_A to ettringite), 725 J/g (C_4_AF) [40]. When the reported degree of hydration is negative, they are assumed to be zero. Based on these calculations and the results from isothermal calorimeter, after 1 day of hydration the heat release associated to slag hydration was negative (−10 to −20 J/g_-slag_). This can be attributed to a combination of negligible slag hydration after 1 day of hydration [10] and the unrecorded heat of hydration in the first 45 min in calorimeter (due to the time required for paste preparation and stabilization of the temperature within the calorimeter).

On the other hand, after 7 days of hydration, the heat release from slag was estimated to be 86.7, 143.8, 100.4 and 212.1 J/g_-slag_ for III_10, III_20, NS_10 and NS_20 respectively. This further validates the conclusion that with the addition of Na_2_SO_4_ and the increase in curing temperature from 10 °C to 20 °C, more slag reacted within 7 days. With the help of enthalpy of slag hydration, it would be straightforward to quantitatively determine the degree of hydration of slag. Currently, there is no agreed value for enthalpy of slag hydration, and previous studies estimated this to be 404 to 521 J/g [64], 462 J/g [65], 530 J/g [66]. Such a large variation is expected due to variations in the slag chemistry, complex multiple reactions involved and accuracy limits for quantitative measurements.

From the current study, based on the degree of hydration of slag determined from XRD and heat release measured from calorimeter, the enthalpy of associated heat release was calculated to be 498, 510, 587 and 625 J/g_-slag_ for III_10, III_20, NS_10 and NS_20 respectively. The error was estimated to be ~±60 J/g_-slag_. The enthalpy of slag hydration appeared to be higher in the presence of Na_2_SO_4_. Although this was attributed to the enhanced production of ettringite, a further systematic study with more datapoints is required to validate such claims in the next step of research.

### 4.3. Strength Development

While there is a clear improvement in mortar strength in PC-fly ash- Na_2_SO_4_ systems as reported in [39,67], this is arguable for PC-slag-Na_2_SO_4_ systems studied here. Strength improvement in early-ages (until 7 days) with the addition of Na_2_SO_4_ was consistent in the literature, but there were some disagreements in the later-age strength. Zhao et al. [35] reported that the compressive strength is higher with Na_2_SO_4_ activation of 5% in 50:50 PC-slag blends. The effect of Na_2_SO_4_ was found to be negligible in the later ages with 2% addition in [31] and 4% addition in [36]. Fu et al. [33] reported considerable decrease of around ~10% at 28 days with 1–3% of Na_2_SO_4_. Mota et al. [30] showed that depending on the type of slag, the later age strength with Na_2_SO_4_ was either comparable or significantly lower with respect to the reference white cement-slag blend. It must be inferred that the effectiveness of Na_2_SO_4_ to activate PC-slag blends depends on the properties of the slag used or PC minerology or both, particularly at later ages. Those factors that affect the later age strength development is currently unknown and needs to be studied further.

Looking back at the results of compressive strength tests (Figure 2), although it can be generalized that the addition of Na_2_SO_4_ and an increase in curing temperature contribute to strength improvement, the reasons for this trend are different at different ages. After 1 day of hydration, strength was influenced primarily by the alite hydration, as the degree of reaction from other clinker phases and slag was negligible. By 7 days, alite was almost fully reacted for all the systems, and the main difference here was the degree of hydration of slag. By 28 and 90 days, the degree of hydration of clinker was similar for all the systems. Degree of slag reaction was lower for III_10, while other systems had very similar degree of hydration. The main factor, which contributed to the later age strength (28 and 90 days), was found to be the ettringite content. The higher sulphate content originated from the additional Na_2_SO_4_ in NS systems produced more ettringite. The low density of this hydrate ensured that it filled the microstructure and reduced the porosity of the NS systems.

## 5. Conclusions

The present study investigated the effect of Na_2_SO_4_ on the strength development, hydration and microstructure development of a commercial CEM III/B 42.5 cement cured at 10 and 20 °C. The main conclusions from the study are:

- Compressive strength is enhanced with the addition of Na_2_SO_4_ at all studied ages (1, 7, 28 and 90 days) and curing temperatures.
- There is a significant improvement in the degree of hydration of alite after 1 day of hydration with Na_2_SO_4_, but the overall degree of reaction of clinker is very similar at later ages.
- Addition of Na_2_SO_4_ boosts the degree of hydration of slag at early ages (up to 7 days) at both 10 and 20 °C, but in the later ages, higher degree of hydration with Na_2_SO_4_ was only found for the mixture cured at 10 °C.
- Without the addition of Na_2_SO_4_, a clear peak for C_3_A hydration is visible, but with its addition, although a peak for C_3_A is not present, there is a clear hydration peak of slag.
- The enthalpy of slag hydration was found to be 498–510 J/g_-slag_ and 587–625 J/g_-slag_ with and without Na_2_SO_4_ addition respectively. The higher enthalpy with Na_2_SO_4_ should be due to the higher enthalpy of formation of ettringite.
- Ettringite and C-S-H are the major hydrate phases in these systems with and without Na_2_SO_4_, while a significant increase in the amount of ettringite was found for the Na_2_SO_4_ systems.
- Microstructure is densified and the overall porosity decreases with the addition of Na_2_SO_4_
- The improvement of strength with the addition of Na_2_SO_4_ is attributed to the increased reaction of cement phases and slag at early ages, while at later ages, it is due to the higher ettringite content.

Further studies are required to screen the suitable slag/cement/Na_2_SO_4_ compositions to achieve strength improvement at later ages, and the durability of such systems needs to be further investigated. The high ettringite content in NS mixes points out that such mix compositions would not be suitable for curing at very high temperatures due to the risk of delayed ettringite formation. It is not yet very clear how these compositions will perform against external sulphate attack.

## Figures and Tables

**Table 1:** Mortar mix compositions and flow values as determined with flow table test.

MIX ID	CEM III (g)	Sand (g)	Solution (g)	[Na_2_SO_4_]	*w/c* Ratio	S/c Ratio	Mortar Flow (mm)
III	450	1350	225	-	0.5	0.5	130 ± 2
NS	450	1350	249	0.75 M	0.5	0.55	146 ± 3

**Table 2:** Chemical composition determined from XRF, mineralogical composition from XRD and physical properties of the cement used.

Chemical Composition		Mineralogical Composition		Physical Properties	
CaO	48.5	C_3_S	18.0	Blaine	5024 cm^2^/g
SiO_2_	29.4	C_2_S	2.3	Specific gravity	2.99 g/cm^3^
Al_2_O_3_	8.4	C_3_A	2.0		
MgO	5.4	C_4_AF	2.0	Selective dissolution residue	69.4%
SO_3_	5.2	Bassanite	1.2		
Fe_2_O_3_	1.0	Anhydrite	1.9		
K_2_O	0.6	Quartz	0.3	Particle size	
TiO_2_	0.6	Calcite	1.0	d10 (µm)	1.4
Na_2_O	0.3	Arcanite	1.2	d50 (µm)	8.8
MnO	0.2	Syngenite	0.9	d90 (µm)	21.0
P_2_O_5_	0.1	Amorphous	69.1		

**Table 3:** Elemental ratios and oxide composition of anhydrous slag from SEM-EDX analysis.

Elemental Ratios (wt%)			Oxide Composition (wt%)			
Si/Ca	Al/Si	Mg/Si	CaO	SiO_2_	Al_2_O_3_	MgO
0.52 ± 0.07	0.35 ± 0.03	0.26 ± 0.04	43.3 ± 3.5	34.5 ± 3.7	10.5 ± 1.0	6.6 ± 1.3

**Table 4:** Degree of hydration (%) and absolute weight (g/100 g anhydrous) in brackets of different clinker phases determined from XRD/Rietveld.

	Age (Days)	C_3_S	C_2_S	C_3_A	C_4_AF
CEM III/B 42.5N	0	0 (18.0)	0 (2.3)	0 (2.0)	0 (2.0)
III_10	1	37.4 (11.3)	18.4 (1.9)	10.6 (1.8)	−7.7 (2.2)
	7	92.5 (1.3)	−11.9 (2.6)	75.0 (0.5)	19.3 (1.6)
	28	93.8 (1.1)	4.9 (2.2)	100 (0)	17.1 (1.7)
	90	96.3 (0.7)	28.4 (1.6)	100 (0)	62.7 (0.7)
III_20	1	70.3 (5.3)	0.6 (2.3)	17.0 (1.7)	38.6 (1.2)
	7	94.7 (1.0)	−14.7 (2.6)	94.4 (0.1)	40.4 (1.2)
	28	93.3 (1.2)	−13.3 (2.6)	100 (0)	51.0 (1.0)
	90	98.0 (0.7)	35.7 (1.5)	100 (0)	71.6 (0.6)
NS_10	1	58.7 (7.4)	−13.9 (2.6)	30.2 (1.4)	6.9 (1.9)
	7	95.5 (0.8)	−19.0 (2.7)	78.5 (0.4)	17.6 (1.6)
	28	96.8 (0.6)	−2.0 (2.3)	65.6 (0.7)	27.5 (1.5)
	90	97.8 (0.4)	29.8 (1.6)	80.2 (0.4)	49.8 (1.0)
NS_20	1	83.0 (3.1)	−4.4 (2.4)	53.1 (0.9)	35.1 (1.3)
	7	95.1 (0.9)	2.5 (2.2)	87.6 (0.2)	45.0 (1.1)
	28	95.2 (0.9)	4.6 (2.2)	76.1 (0.5)	33.6 (1.3)
	90	96.3 (0.7)	42.8 (1.3)	100 (0)	63.9 (0.7)

**Table 5:** Elemental ratios (atomic) for different mixes at 28 days using SEM-EDX analysis.

	III_10	III_20	NS_10	NS_20
Si/Ca	0.54 ± 0.08	0.54 ± 0.13	0.59 ± 0.06	0.59 ± 0.07
Al/Ca	0.17 ± 0.03	0.18 ± 0.05	0.17 ± 0.02	0.17 ± 0.03
S/Ca	0.08 ± 0.03	0.06 ± 0.03	0.11 ± 0.03	0.11 ± 0.02
Al/Si	0.31 ± 0.05	0.32 ± 0.06	0.31 ± 0.05	0.30 ± 0.05
Mg/Si	0.18 ± 0.07	0.16 ± 0.09	0.23 ± 0.09	0.23 ± 0.12
Na/Ca	0.01 ± 0.01	0.02 ± 0.01	0.12 ± 0.05	0.08 ± 0.05

## Data Availability

Not Applicable.
